# Obituary

**Published:** 1866-11

**Authors:** 


### OBITUARY.

In the *Nevada* (California) *Daily Transcript* of Sept 1, 1866, we read the following:

DIED —In this city, Sept. 1st, 1866, Dr. EDWIN G. MEEK, aged 44 years.

Dr. Meek was co-editor of this Journal, with Dr. John Evans, from April, 1849, to February, 1851. He had previously contributed several interesting papers for the Journal, among which was one on "The Physical Condition of the Aborigines, with an Account of their Practice of Medicine," written while he was stationed at the Choctaw Agency, West Arkansas.

In his valedictory of February 15, 1851, on resigning his position as editor of this Journal, he states that he is about to leave Chicago for the practice of medicine on the Pacific coast.

The success of the Journal during the period of his connection with it, depended, in a great measure, upon the labors of Dr. Meek.

The deceased was regarded as a man of fine abilities and excellent education, and during his residence in Chicago, received the confidence and esteem of the profession.

---

*A Doctor's "Line:" Illustrating a New Mode of Getting a Prescription from the Apothecary's.*—In a not very populous district in the neighborhood of the Scottish town of Dumfries, there resides a carter, named Brown, with his wife and mother. One day lately the old lady took alarmingly ill; the son hurried to the town and returned with a physician. The patient was examined, and a piece of paper called for, on which to note the remedies to be administered. Not a suitable piece of paper was, however, to be found in this isolated domicile, and singular enough, the doctor had not a scrap in his possession. "Have you a piece of chalk, then?" somewhat gruffly inquired the M. D. He was answered in the affirmative, provided with the article, wrote the prescription out on the door, and, taking leave, told his employer to get the parish schoolmaster to transcribe it. Brown, however, was not disposed to put himself under obligation to even such a genial personage as the village dominie, and, though he may not have heard of Mr. Smiles' "Self-Help," he determined on a course that showed he was at least familiar with the adage, "He is best served who serves himself." The fastenings of the hinges were immediately removed, the door taken down, laid on a barrow, and wheeled into town with all possible haste. Arrived at Dumfries, he strode into an apothecary's shop with the door on his shoulder, and the astonishment of the knight of the pestle and mortar, when it was placed on the counter with the words, "There's a line from Dr.——," may be better imagined than described. Apothecaries, however, are not quite so particular as bankers as to what they honor, and our friend received his medicine without being subjected to many queries.

---

*Sign of the Times.*—In one of the busy thoroughfares of Chicago may be seen the following dubious announcement: "Cholera cured without fail."

